# TLR2 Signaling in Myeloproliferative Neoplasms: Pathophysiological Roles, Mutation-Dependent Heterogeneity, and the Differential Efficacy of Ruxolitinib

**DOI:** 10.3390/ijms27209083

**Published:** 2026-10-13

**Authors:** Akriti Pokhrel, Narek Hakobyan, Jen Chin Wang

**Affiliations:** Department of Hematology and Medical Oncology, Brookdale University Hospital Medical Center, One Brooklyn Health, Brooklyn, NY 11212, USA; akriti.pp@gmail.com (A.P.); hakobyan.narek77@gmail.com (N.H.)

**Keywords:** myeloproliferative neoplasm (MPN), polycythemia vera (PV), essential thrombocythemia (ET), myelofibrosis (MF), Toll-like receptor-2 (TLR2), cytokine, ruxolitinib, thrombosis, JAK/STAT, NF-KB

## Abstract

Toll-like receptor 2 (TLR2) is a versatile pattern-recognition receptor of the innate immune system that recognizes both pathogen-associated molecular patterns (PAMPs) and endogenous danger-associated molecular patterns (DAMPs). Philadelphia chromosome-negative myeloproliferative neoplasms (MPNs)—clonal hematopoietic stem-cell disorders that include essential thrombocythemia (ET), polycythemia vera (PV), and myelofibrosis (MF)—are characterized by persistent inflammation and an increased risk of thrombosis. Dysregulated TLR2 signaling has emerged as a potentially important mediator of this chronic thrombo-inflammatory state. Findings from our group and others suggest that TLR2 may have a particularly prominent role in promoting inflammatory cytokine production, platelet–leukocyte interactions, endothelial dysfunction, and thrombotic complications in MPNs. Our findings are also consistent with evidence that NF-κB-mediated proinflammatory signaling, in addition to constitutive JAK/STAT activation, contributes substantially to MPN pathogenesis. In this review, we provide a comprehensive overview of TLR2 biology and examine its contribution to the thrombo-inflammatory milieu of MPNs, with particular emphasis on its potential utility as a biomarker and a therapeutic target. Ruxolitinib, a JAK1/2 inhibitor, markedly reduces symptom burden and splenomegaly in JAK2-mutant MPNs, particularly PV and MF; however, it has limited efficacy in CALR-mutant or triple-negative ET. This differential therapeutic response may reflect divergent downstream signaling pathways and may also be related to dysregulated TLR2 signaling. Furthermore, hyperactive TLR2 signaling in MPNs may help explain the incomplete ability of ruxolitinib to suppress thrombo-inflammatory responses. Therefore, combined TLR2-targeted therapy and ruxolitinib may represent a more effective therapeutic strategy for future MPN trials. Overall, however, we consider the TLR2-mediated thrombo-inflammatory response in MPNs to be an amplifier of inflammation rather than a primary disease driver.

## 1. Introduction

Myeloproliferative neoplasms (MPNs) are clonal hematopoietic stem cell disorders defined by overproduction of one or more mature blood cell lineages. The three classical Philadelphia chromosome-negative MPNs—ET, PV, and primary MF—share recurrent somatic driver mutations in JAK2, CALR, or MPL, each activating the JAK-STAT signaling pathway to varying degrees and through distinct mechanistic routes [1]. Hyperactive JAK2 signaling is a unifying downstream feature across MPN phenotypes, and we have previously reported that TLR2 is one of the primary inflammatory signaling pathways in MPN diseases [2]; therefore, herein, we review the role of TLR2 in inflammatory signaling pathways in MPNs.

Thrombosis remains the leading cause of morbidity and mortality in ET and PV, driven by an activated prothrombotic phenotype affecting platelets, leukocytes, and endothelial cells [3]. In MF, progressive bone marrow fibrosis, ineffective hematopoiesis, anemia, and constitutional symptoms dominate, with transformation to acute leukemia representing the most serious complication [4]. A critical insight from the past decade is that inflammation is not merely a bystander in MPNs but an active driver of disease pathogenesis, sustained by a self-reinforcing loop in which clonal blood cells release proinflammatory mediators that, in turn, activate the MPN clone, amplifying cellular activation, coagulation, and fibrosis [5,6].

Toll-like receptors (TLRs) are transmembrane pattern-recognition receptors (PRRs) constituting a first line of defense against microbial invasion by recognizing conserved molecular motifs on pathogens. Crucially, TLRs also sense endogenous DAMPs released from damaged or dying cells, positioning them as pivotal sensors of sterile inflammation [7]. TLR2 forms functional heterodimers with TLR1 (which recognizes triacylated lipopeptides) or TLR6 (which recognizes diacylated lipopeptides) and is expressed on platelets, monocytes, neutrophils, megakaryocytes, and hematopoietic progenitors. Its activation initiates MyD88-dependent NF-κB and MAPK/ERK signaling cascades that drive proinflammatory cytokine production, cellular activation, and coagulation (Figure 1) [7,8,9].

Multiple endogenous TLR2 ligands have been identified in MPN patients—including extracellular histones and DNA from neutrophil extracellular traps (NETs), heat shock protein 27 (Hsp27), and EDA-fibronectin—providing a mechanistic basis for constitutive TLR2 stimulation in the absence of overt infection [10,11]. Platelets from ET patients display selective TLR2 hyperresponsiveness [12], and monocytes from MF patients exhibit exacerbated TLR2- and TLR4-triggered cytokine production [13,14], together creating a cellular environment of sustained immune activation that fuels the thrombo-inflammatory cycle. Therefore, findings from our study [2] and the study by Oyarzún et al. [12] suggest that TLR2 may play a more prominent role than TLR4 in the thrombo-inflammatory pathways associated with MPNs. Previous studies have demonstrated important roles of TLR4 and RAGE in S100A8/A9-mediated inflammatory responses, but TLR2 signaling was not investigated in those studies [15,16]. Thus, TLR4 is also likely to contribute to MPN-associated inflammation, although its relative importance and pathway-specific functions require further investigation.

This review thoroughly examines TLR2 signaling in MPNs, explores how driver mutations shape TLR2-dependent inflammation, and proposes frameworks for understanding and exploiting mutation-dependent heterogeneity in TLR2 biology for therapeutic benefit.

## 2. TLR2 Biology: Structure, Signaling, and Cellular Expression

### 2.1. Structural Features and Dimerization (Figure 1)

TLR2 is a type I transmembrane glycoprotein composed of an extracellular leucine-rich repeat (LRR) domain responsible for ligand recognition, a single transmembrane domain (TMD), and a cytoplasmic Toll/IL-1 receptor (TIR) homology domain that recruits downstream adaptors [7]. Unlike TLR4, which functions as a homodimer, TLR2 achieves ligand specificity through obligate heterodimerization: it pairs with TLR1 to recognize triacylated lipopeptides found in Gram-negative bacteria and mycoplasma, and with TLR6 to sense diacylated lipopeptides from Gram-positive bacteria. TLR6 lacks a hydrophobic binding pocket for the third lipid chain, conferring differential recognition of di- versus triacylated ligands [7]. The synthetic lipopeptide Pam3CSK4, a prototypical TLR2/TLR1 agonist, has been widely employed in experimental MPN studies to specifically activate this receptor complex [8,12,14].

TLR2 dimerization is coordinated through ligand binding to the extracellular domain, triggering lateral movement of receptor monomers and conformational changes transmitted through the TMD to the intracellular TIR domain. Notably, the TMD itself participates in receptor assembly: peptides derived from the TLR2 TMD region (TLR2-p) can physically interact with TLR1, TLR2, and TLR6 within the membrane, thereby inhibiting ligand-induced dimerization and downstream signaling [9]. This principle has been validated therapeutically in murine colitis models, where TLR2-p administration selectively neutralized the proinflammatory potential of infiltrating Ly6Chi monocytes without impairing their recruitment—a distinction with important therapeutic implications in inflammatory diseases, including MPNs [9].

### 2.2. Downstream Signaling Pathways

Upon ligand-induced dimerization, TLR2 recruits the adaptor protein MyD88 directly and, for some ligands, through the bridging adaptor TIRAP (MAL). MyD88 engagement initiates a signaling cascade through IRAK4, IRAK1/2, and TRAF6, culminating in the formation of a ‘myddosome’ complex. TRAF6 activates TAK1, which phosphorylates IKKβ to liberate NF-κB from its inhibitor IκBα, allowing nuclear translocation and transcription of proinflammatory genes including TNF-α, IL-1β, IL-6, IL-8, and MCP-1 [5]. In parallel, TAK1 activates MAPK cascades, including p38, JNK, and ERK1/2, which contribute to cytokine production, cellular activation, and, crucially in platelets, granule secretion and degranulation [7,12]. Unlike TLR4 (Figure 1), TLR2 does not engage the TRIF-dependent signaling pathway and therefore does not induce type I interferons. This restricts TLR2’s primary downstream output to NF-κB and MAPK activation. In platelets, TLR2 engagement by Pam3CSK4 triggers phosphoinositide 3-kinase (PI3K) activation and ERK1/2 phosphorylation, leading to P-selectin surface translocation, CD40L exposure, dense granule exocytosis (CD63 expression), and RANTES (CCL5) secretion [12,15]. Critically, enhanced TLR2-mediated granule secretion in ET platelets is not correlated with increased GPIIbIIIa (PAC-1) activation, indicating that TLR2 selectively amplifies the inflammatory rather than the classical hemostatic platelet response—a pharmacologically exploitable distinction [12].

In monocytes and macrophages, TLR2 signaling drives robust production of IL-1β, IL-6, IL-12, and IL-23 [5]. In the context of *H. pylori* infection, TLR2 specifically mediates IL-6 and IL-1β secretion by bone marrow-derived macrophages through a MyD88-dependent pathway, while TLR4 is preferentially responsible for IL-12 and IL-10 production—illustrating functional specificity among TLR family members that is relevant to the cytokine profiles observed in MPNs [8].

### 2.3. TLR2 Expression in Hematopoietic Cells

TLR2 is expressed on the surface of platelets, monocytes, neutrophils, dendritic cells, and megakaryocytes, and is also present on CD34+ hematopoietic progenitor cells [2,7]. The broad cellular distribution of TLR2 across the MPN clone means that TLR2-mediated inflammation can operate simultaneously at multiple levels: promoting progenitor expansion, enhancing megakaryocyte maturation, activating mature platelets, and driving monocyte cytokine production.

In megakaryocytes, TLR2 activation by Pam3CSK4 induces NF-κB, ERK-MAPK, and PI3K/AKT pathway activation, increases transcription factors related to megakaryocyte maturation (mTOR, GATA-1, NF-E2), and upregulates CD41 and CD61 surface markers—indicating that TLR2 can directly promote thrombopoiesis [7,16]. TLR2-deficient mice have lower platelet counts than wild-type animals, confirming a physiological role of TLR2 in platelet production [7,16]. In the inflammatory context of MPNs, where endogenous TLR2 ligands are chronically elevated, constitutive TLR2 stimulation may thus contribute not only to platelet activation but also to the reactive thrombocytosis that characterizes ET and MF [7,12].

## 3. TLR2 in MPN Pathogenesis

### 3.1. Endogenous TLR Ligands in MPNs: The DAMP Environment

A prerequisite for understanding TLR2’s role in MPNs is recognizing that multiple endogenous TLR2 and TLR4 ligands are chronically elevated in MPN patients, establishing a DAMP-rich milieu that constitutively activates TLR signaling in the absence of infection [10,11,12,14]. This sterile inflammatory state mirrors conditions seen in autoimmune disorders, atherosclerosis, and cancer, where DAMPs drive pathological TLR activation [7].

Neutrophil extracellular traps (NETs) and their associated histone/DNA complexes represent major endogenous TLR2 and TLR4 ligands in MPNs. Circulating nucleosomes and NET remnants are elevated in MPN patients, correlating with disease activity and thrombotic risk [10]. Extracellular histones, released during NET formation or cellular necrosis, activate both TLR2 and TLR4 on platelets and monocytes, triggering thrombin generation through platelet-dependent mechanisms [10,17].

Heat shock protein 27 (Hsp27) is an endogenous TLR ligand elevated in MPNs, acting as a JAK2-STAT5 partner whose levels correlate with JAK2V617F allele burden. EDA-fibronectin (EDA-FN), a cellular fibronectin isoform, engages TLR4 on megakaryocytes, sustaining megakaryocyte expansion and inflammation in bone marrow fibrosis through profibrotic IL-6 release—directly linking TLR4 signaling to MF progression [11]. The finding that tasquinimod, an S100A9 inhibitor, ameliorates myeloproliferation and bone marrow fibrosis in JAK2V617F murine models further underscores the therapeutic importance of the DAMP/TLR axis [18].

Most recently, two prototypical DAMPs—HMGB1 and S100A8/A9—have been identified as significantly elevated in MF patients [14]. HMGB1 levels were higher in MF patients compared to controls, rising progressively with disease stage as stratified by both the DIPSS and MIPSS70 prognostic systems, and correlating with adverse clinical features including anemia, constitutional symptoms, and inferior overall survival [14]. S100A8/A9, a heterodimeric alarmin highly expressed in monocytes and neutrophils, was similarly elevated in MF and showed a close correlation with monocyte and leukocyte counts, suggesting active cellular release as a major source [14]. Both DAMPs signal through TLR4, and HMGB1 additionally engages TLR2, providing direct mechanistic links between DAMP elevation and TLR-mediated monocyte hyperactivation in MF [14]. The correlation of plasma IL-1β and IL-6 with DAMP levels raises the possibility of a positive feedback loop in which DAMPs drive TLR-dependent cytokine production that further amplifies DAMP release through NLRP3 inflammasome activation and pyroptotic cell death [14,19].

### 3.2. TLR2-Mediated Platelet Hyperresponsiveness in ET

The seminal study by Marín Oyarzún et al. [12] established that TLR2 ligation leads to selectively exacerbated platelet responses in ET patients. Using Pam3CSK4 as a specific TLR2/TLR1 agonist, this study demonstrated that ET platelets show significantly increased surface translocation of α-granule-derived P-selectin and CD40L compared to healthy controls than TLR4 upon TLR2 stimulation, whereas the response to the classic hemostatic agonist TRAP-6 (PAR-1 agonist) was impaired or preserved.

P-selectin translocation mediates platelet binding to PSGL-1 on neutrophils and monocytes, initiating platelet–leukocyte cross-talk [12]. Engagement of CD40L with its endothelial receptor CD40 upregulates adhesion molecules and proinflammatory cytokines, contributing to endothelial activation. In ET, TLR2-triggered CD40L exposure was significantly more enhanced than with TLR4, suggesting that TLR2 activation could amplify platelet–endothelial interactions beyond what is achieved through classical hemostatic pathways [12]. Dense-granule-derived CD63 was also increased following TLR2 stimulation compared with TLR4 [1].

Beyond adhesion molecules, TLR2 and TLR4 stimulation both triggered significantly higher RANTES (CCL5) secretion from ET platelets compared to controls, while von Willebrand factor (VWF) release was not enhanced [12]. RANTES is a potent chemoattractant that drives monocyte recruitment to vascular walls—a critical step in atherogenesis—and cooperates with PF4 to prime NET formation [12,20]. The selective upregulation of RANTES without concomitant VWF elevation suggests differential α-granule secretion, consistent with the concept of specialized granule subpopulations that are selectively released based on the nature of the activating stimulus. Despite elevated platelet RANTES secretion, circulating RANTES in ET patients was rarely elevated, implying that platelet-released RANTES mediates local rather than systemic inflammation [12].

TLR2 and TLR4 expression levels on the platelet surface did not differ between ET patients and controls, excluding receptor overexpression as the mechanism of TLR2 hyperresponsiveness [12]. Rather, ERK1/2 phosphorylation was constitutively elevated in resting ET platelets from both JAK2-positive and CALR-positive patients, suggesting that hyperactivated ERK1/2—a convergence point of JAK2 signaling and TLR2 downstream signaling—represents a shared mechanistic node that amplifies TLR2 responses irrespective of driver mutation type [12]. The fact that Pam3CSK4 increased, but not due to LPS, again suggests that TLR2 is the prime inducer for the platelet-associated thrombo-inflammatory response, mechanistically explaining the observed functional hyperresponsiveness [12].

### 3.3. Platelet–Neutrophil Aggregates: TLR2 as an Amplifier of Thromboinflammation

Platelet–neutrophil aggregates (PNAs) are well-established markers of in vivo platelet activation in MPNs and have been mechanistically linked to thrombosis development. In MPN patients, we found that TLR2 agonists have a stronger response than TLR4 agonists [2]; moreover, in PNA, circulating PNAs are elevated at baseline and increase further upon TLR2 and TLR4 stimulation, and TLR2 appears to have a stronger response than TLR4. Oyarzun [12] also found that TLR agonists had a stronger response than TLR4 agonists, suggesting that TLR exhibits a stronger response than TLR4 to PNA formation.

The functional consequences of enhanced TLR-triggered PNA formation are profound. Activated neutrophils within platelet–neutrophil complexes produce reactive oxygen species (ROS), which are elevated in MPNs, and release NETs—structured chromatin-enzyme scaffolds that activate coagulation and damage the vascular endothelium [10]. In turn, NET-derived histones and DNA constitute endogenous TLR2/TLR4 ligands, creating a positive feedback amplification loop: TLR2 activation promotes PNA formation, PNA formation drives NET release, and NET-derived DAMPs re-stimulate TLR2 and TLR4, further amplifying thromboinflammation [10,12]. This self-sustaining cycle may explain why the MPN thrombo-inflammatory state persists even in well-treated patients with normalized blood counts.

Notably, the relationship between platelets and leucocytes in TLR-mediated responses is not exclusively proinflammatory. Hally et al. [21] demonstrated in healthy subjects that platelets act as complex immunomodulators, differentially regulating leucocyte responses to TLR stimulation in a TLR agonist-specific manner. In platelet–leucocyte cocultures, platelet coculture reduced neutrophil CD66b expression in response to LPS (TLR4), Pam3CSK4 (TLR2/1), and FSL-1 (TLR2/6) stimulation, and attenuated granulocyte elastase secretion in response to low doses of all three agonists. In response to LPS, platelets reduced IL-6, TNF-α, and MIP-1β production while increasing IL-10 production by peripheral blood mononuclear cells (PBMCs)—indicating a shift toward anti-inflammatory cytokine output. In contrast, platelet coculture did not alter PBMC cytokine production in response to Pam3CSK4 (TLR2/1), while FSL-1 (TLR2/6) responses were modulated in a mixed fashion, with increased IL-6 and IL-10 but reduced TNF-α. These findings challenge the notion that platelets act solely in a proinflammatory manner and suggest that, in MPNs, where platelet activation and leukocyte priming co-exist, the net inflammatory outcome of TLR2 stimulation may reflect a complex, bidirectional platelet–leucocyte regulatory interaction. The platelet-dampening effect on leucocyte TLR responses may represent a partially preserved homeostatic mechanism in MPNs, though one likely overwhelmed by the constitutively elevated DAMP environment and dysregulated downstream signaling in the disease state.

### 3.4. TLR2-Triggered Monocyte Hyperactivation in Myelofibrosis

Monocytes are central mediators of MF inflammation, constituting a major source of proinflammatory cytokines, including IL-1β, IL-6, TNF-α, IL-8, and TGF-β, that drive bone marrow fibrosis [13]. Multiple studies have demonstrated monocyte hyperactivation at baseline in MF, reflected by elevated CD11b expression, tissue factor surface exposure, and heightened cytokine gene transcription [13,14]. Critically, MF monocytes show preserved or even exacerbated functional responses to TLR2 (Pam3CSK4) and TLR4 (LPS) stimulation at low and intermediate agonist concentrations, indicating that these cells are primed rather than anergic [14].

This priming is mechanistically consistent with the elevated DAMP environment in MF circulation: HMGB1 and S100A8/A9 binding to TLR2/TLR4 could lower the activation threshold of monocytes through NF-κB pre-activation, rendering them hyperresponsive to subsequent TLR stimulation [14]. The incubation of healthy monocytes with patient plasma samples containing elevated HMGB1 and/or S100A8/A9 reproduced the IL-1β upregulation seen in primary MF monocytes, providing direct experimental evidence for DAMP-mediated monocyte priming [14]. In MF, monocytes also exhibit impaired negative regulation of TLR signaling, leading to excessive TNF-α release upon TLR4 and TLR7/8 stimulation [22]. Fisher et al. [13] demonstrated that MF monocytes produce exaggerated amounts of several cytokines upon TLR1/2 and TLR7/8 ligation through NF-κB-dependent mechanisms, collectively establishing TLR2 as a pathogenic amplifier of inflammation in MF (Figure 2).

### 3.5. TLR2 Expression Across MPN Subtypes and Its Role in Leukocyte–Platelet Interactions and Thrombosis

We [2] conducted a comprehensive study of TLR2 expression in 119 Ph(−) MPN patients (51 ET, 37 PV, 31 MF) compared to 21 normal controls, providing direct quantification of TLR2 levels across disease subtypes. TLR2 was significantly elevated in PV (mean MFI 358.5 ± 26.01; *p* < 0.001) and ET (292.3 ± 13.13; *p* = 0.009) relative to controls (220.6 ± 9.8), with PV exhibiting significantly higher TLR2 levels than ET (*p* < 0.05). Notably, MF patients did not demonstrate elevated TLR2 relative to controls, and none of the post-ET MF, post-PV MF, or PMF patients had elevated TLR2 values; meanwhile, TLR4 values were not significantly more elevated in either PV, ET, or MF patients than in controls, and only when ET, PV, and MF patients were grouped together as MPN patients were the results statistically significant. This is consistent with a model in which TLR2-driven inflammation characterizes early-stage MPN (ET/PV) and transitions to ROS-dependent mechanisms in advanced disease, and TLR2 is more likely than TLR4 to play a significant role in the thrombo-inflammatory response in MPNs. In TLR2, overexpression was identified across multiple cell populations including CD34+, CD3+, CD14+, CD71+, and CD20+ cells, indicating that TLR2 upregulation simultaneously encompasses stem cells and immune effector cells.

In TLR2-elevated MPN patients, monocyte-derived dendritic cells (mdDCs) stimulated with the TLR2 agonist Pam3CSK4 secreted significantly higher levels of TNF-α and IL-8 than those from patients with normal TLR2. Plasma IL-1β was also elevated in patients with elevated TLR2, and other cytokines trended upward, with non-significance likely attributable to sample size. TLR2 expression levels correlated with JAK2 V617F mutation status (patients with JAK2 mutations had higher TLR2 than controls (*p* = 0.007)) and with leukocytosis (TLR2 was significantly higher in patients with WBC > 10 × 109/L (*p* = 0.03)), mirroring established clinical risk factors for thrombosis in MPNs.

Leukocyte–platelet interaction (LPI) assays in this cohort further established the functional relevance of TLR2 to thrombosis. Unactivated-MPN patients showed greater baseline LPI than controls (13.01 ± 1.12% vs. 5.01 ± 0.97%; *p* < 0.05), consistent with intrinsic platelet hyperactivation. Upon stimulation with the TLR2 agonist PM3CSK4, MPN patients exhibited significantly greater LPI than with the TLR4 agonist LPS (21.39 ± 1.07 vs. 12.12 ± 2.16; *p* < 0.01). Critically, the TLR2 inhibitor C29 significantly suppressed LPI in both unactivated and activated MPN samples, whereas the TLR4 inhibitor C34 had no inhibitory effect—directly implicating TLR2, rather than TLR4, as the primary driver of leukocyte–platelet aggregation in MPNs. The clinical correlate of these functional findings was a significantly higher thrombosis incidence in patients with elevated TLR2 (29%) compared to those with normal TLR2 levels (11%), with elevated-TLR2 patients also showing higher WBC counts (*p* = 0.02) [2]. Additionally, plasma S100A9 was significantly elevated in PV and MF (but not ET) relative to controls, and ROS were markedly increased only in MF—supporting a disease progression model in which TLR2-driven inflammation in early MPNs (ET/PV) precedes S100A9-mediated ROS generation and DNA damage, which induces a transformation to MF or leukemia.

## 4. Mutation-Dependent Heterogeneity in TLR2 Signaling

### 4.1. JAK2V617F: ERK1/2 as the Mechanistic Bridge

JAK2V617F is a gain-of-function point mutation (Val617Phe) in the pseudokinase domain of JAK2 that constitutively activates JAK-STAT signaling, present in approximately 95% of PV patients, 50–60% of ET patients, and 50–60% of MF patients [1]. Beyond JAK-STAT activation, JAK2V617F constitutively phosphorylates ERK1/2—a kinase whose activity is essential for TLR2-mediated platelet granule secretion and cellular activation. ERK1/2 hyperactivation has been documented in JAK2V617F-mutant cell lines and MPN progenitors [23]; data from ET platelet studies confirm that this extends to mature platelets and represents a mutation-linked priming mechanism for TLR2 hyperresponsiveness [12].

The convergence of JAK2V617F downstream signaling and TLR2 downstream signaling on the ERK1/2 node creates a mechanistic synergy: constitutively activated ERK1/2 lowers the threshold for TLR2-mediated inflammatory responses, such that even subthreshold TLR2 stimulation can trigger robust platelet activation and monocyte cytokine production in JAK2-positive patients [12,24]. This synergy is particularly relevant given the chronic DAMP exposure in MPN circulation—DAMPs that would normally elicit only modest TLR2 responses are amplified by JAK2-driven ERK priming to produce pathologically exaggerated inflammatory outputs [14].

From a therapeutic standpoint, this mechanistic convergence explains why ruxolitinib—by inhibiting JAK1/2 and thereby reducing ERK1/2 phosphorylation—might attenuate TLR2-mediated inflammatory responses in JAK2-positive MPNs. Ruxolitinib’s documented anti-inflammatory effects in PV and MF, including reductions in circulating cytokines and normalization of elevated C-reactive protein [22], may reflect not only direct inhibition of JAK-STAT-driven cytokine production but also indirect dampening of TLR2 hyperresponsiveness through ERK1/2 downregulation.

### 4.2. CALR Mutations: Alternative Signaling Architecture

Calreticulin (CALR) mutations—predominantly type 1 (52 bp deletion) and type 2 (5 bp insertion) frameshift mutations in exon 9—are found in approximately 25–30% of ET patients and 25–35% of MF patients, being mutually exclusive with JAK2V617F [1]. CALR mutations generate a novel C-terminal peptide that aberrantly interacts with and activates the thrombopoietin receptor MPL, leading to constitutive MPL signaling and preferential activation of the JAK2-STAT5 and PI3K/AKT pathways. Crucially, CALR-mutant signaling is substantially less dependent on JAK2 kinase activity and engages ERK1/2 less robustly than JAK2V617F, reflecting the different receptor contexts: CALR-mutant MPL signaling preferentially couples to PI3K/AKT, while JAK2V617F in cytokine receptor complexes more efficiently activates ERK-MAPK cascades [1].

Marín Oyarzún et al. did not specifically distinguish TLR2-triggered platelet responses in JAK2-mutated versus CALR-mutated disease [12], while our study suggests that TLR2 expression is higher in patients with JAK2 mutations than in those with CALR mutations, although this difference did not reach statistical significance, likely because of the limited sample size [2]. A more clinically important functional distinction emerges in ruxolitinib sensitivity. CALR-driven MPL activation may proceed through relatively JAK2-independent mechanisms, including engagement of JAK1 and TYK2, and the PI3K/AKT axis preferentially activated in CALR-mutant cells is not efficiently inhibited by ruxolitinib at therapeutically achievable concentrations [1,4]. In contrast, TLR2 may be more highly expressed and functionally coupled to JAK2-dependent inflammatory signaling in JAK2-mutated disease. This hypothesis could explain why ruxolitinib exerts stronger anti-inflammatory effects in JAK2-mutant MPNs than in CALR-mutant disease, in which TLR2-associated inflammatory responses may be less dependent on JAK2 signaling and, therefore, less responsive to JAK inhibition. However, comprehensive mechanistic and clinical studies are needed to substantiate this hypothesis.

### 4.3. Triple-Negative MPNs: Upstream Diversity with Shared Downstream Inflammation

Approximately 10–15% of ET and MF patients are triple-negative, lacking JAK2, CALR, and MPL mutations [1]. This group is molecularly heterogeneous, with some patients harboring mutations in non-canonical signaling genes (e.g., LNK/So H2B3, CBL, NRAS, KRAS, BRAF). Despite the absence of classical driver mutations, triple-negative MPN patients often present with inflammatory features, elevated cytokines, and thrombotic risk comparable to mutation-positive patients—suggesting that downstream inflammatory pathways such as TLR2 signaling can be engaged through diverse upstream mechanisms [1,6].

In triple-negative MPNs, JAK-STAT hyperactivation is often detectable despite the absence of canonical driver mutations, possibly through alternative activation by growth factor receptor signaling, somatic mutations in other pathway components, or through paracrine cytokine stimulation [1]. Whether TLR2 signaling is dysregulated to the same extent in triple-negative MPNs as in mutation-positive disease, and whether ERK1/2 hyperactivation occurs in this subgroup, remain incompletely characterized questions with important implications for therapeutic targeting.

## 5. Ruxolitinib Efficacy in MPNs: Mechanistic Basis and Limitations in the Context of TLR2 Biology

### 5.1. Ruxolitinib: Mechanism of Action and Clinical Benefits

Ruxolitinib (INCB018424) is a selective, potent inhibitor of JAK1 and JAK2 that competitively blocks ATP binding to the kinase domain. By inhibiting JAK1/2, ruxolitinib reduces constitutive STAT3 and STAT5 phosphorylation, attenuating downstream transcription of genes controlling cell proliferation, survival, and cytokine production [4]. In MF, the COMFORT-I and COMFORT-II trials established ruxolitinib as a disease-modifying agent, achieving ≥35% spleen volume reduction in approximately 40% of patients and significantly improving symptom scores compared to a placebo or the best available therapy [4]. In PV, ruxolitinib achieved superior hematocrit control and symptomatic improvement compared to standard therapy, with approximately 25% of patients achieving complete hematologic remission (RESPONSE trial) [4].

Beyond its direct antiproliferative effects, ruxolitinib exhibits pronounced anti-inflammatory activity. Treatment reduces circulating levels of numerous proinflammatory cytokines, including IL-6, IL-8, IL-2R, TNF-α, and MCP-1, and normalizes elevated C-reactive protein [5,25]. These anti-inflammatory effects likely contribute substantially to the symptomatic benefits of ruxolitinib, as symptom burden in MPNs correlates more closely with cytokine levels than with spleen size or blood counts [5].

### 5.2. Why Ruxolitinib Works Better in JAK2-Positive PV and MF

The superior efficacy of ruxolitinib in JAK2V617F-positive MPNs reflects the direct dependency of disease-driving signaling on JAK2 kinase activity. In JAK2V617F-mutant cells, the mutant JAK2 is constitutively active and directly drives STAT5, ERK1/2, and PI3K/AKT activation [1,23]. Ruxolitinib binds to and inhibits the mutant JAK2 kinase with high potency, directly disrupting the core oncogenic signal. Additionally, because JAK2V617F is present in the dominant clone in most PV patients, ruxolitinib can achieve allele burden reduction—particularly with longer treatment durations—reflecting genuine clonal suppression [4].

In the context of TLR2 biology, JAK2V617F inhibition by ruxolitinib would be expected to reduce ERK1/2 basal phosphorylation in platelets and monocytes, restoring the normal TLR2 activation threshold. This ERK normalization would attenuate TLR2-driven P-selectin expression, RANTES secretion, platelet–neutrophil aggregate formation, and monocyte cytokine production—collectively reducing the thrombo-inflammatory state [12]. In PV specifically, where JAK2V617F is essentially universal and allele burdens are typically high, the convergence of direct clonal suppression, STAT5 inhibition, and ERK/TLR2 axis normalization produces robust clinical benefits. The marked reduction in thrombotic events observed with ruxolitinib in PV aligns with the predicted dampening of TLR2-mediated thrombo-inflammatory activation [4].

In a comparative study previously conducted by our research group, which provides direct evidence supporting this mechanistic framework [24], we proposed that the superior efficacy of ruxolitinib in hydroxyurea-resistant PV compared to ET may be mechanistically explained by the higher TLR2 expression in PV. In our preliminary study of 37 PV patients [26], 51 ET patients, and 21 controls, the mean TLR2 values were 358.5 ± 26.01 (PV), 292.3 ± 13.13 (ET), and 220.6 ± 9.8 (controls), with TLR2 significantly higher in PV than ET (*p* < 0.01). PV patients with elevated TLR2 (TLR2-E) produced significantly more IL-1β plasma cytokine than ET patients with normal TLR2 values (1.55 ± 1.02 vs. 0.93 ± 0.29 pg/mL, *p* < 0.05). In cultured dendritic cells, TNF-α and IL-8 were significantly elevated in PV patients with TLR2-E compared to ET patients with TLR2-N (TNF-α: 47.55 ± 16.78 vs. 15.5 ± 4.0 pg/mL; *p* < 0.05). We postulate that in PV, ruxolitinib operates through the TLR2 inflammatory pathway and its effects on dendritic cell cytokine production, providing a direct molecular explanation for ruxolitinib’s more pronounced anti-inflammatory benefits in PV than in ET. However, we acknowledge that this is indirect evidence, and further studies will be necessary to substantiate this hypothesis.

### 5.3. Why Ruxolitinib Has Limited Efficacy in CALR-Mutant and Triple-Negative MPNs

CALR-mutant MPNs present a fundamentally different therapeutic target profile. The pathogenic CALR mutant protein activates MPL through an extracellular gain-of-function mechanism rather than through JAK2 kinase gain-of-function. While MPL activation does engage JAK2 downstream, the primary signaling outputs preferentially engage JAK2’s kinase partner JAK1 and the PI3K/AKT axis, with relatively less ERK1/2 activation compared to JAK2V617F [1]. Consequently, ruxolitinib—even at full therapeutic doses—achieves only partial suppression of CALR-mutant signaling, leaving residual PI3K/AKT activity that sustains cellular survival and proliferation [1,4].

Multiple clinical observations corroborate this molecular framework. CALR-mutant ET patients have lower thrombotic risk than JAK2-positive ET patients despite comparable platelet counts, possibly reflecting less ERK-dependent platelet activation [1]. CALR-positive MF patients survive for longer than JAK2-positive MF patients in most series, yet show poorer molecular responses (allele burden reduction) to ruxolitinib [4]. From a TLR2 perspective, ruxolitinib’s inability to normalize ERK1/2 activity in CALR-mutant cells means that the TLR2 hyperresponsiveness driven by ERK priming persists despite treatment. The thrombo-inflammatory loop—endogenous DAMPs → TLR2 activation → ERK-dependent platelet/monocyte hyperresponse → more DAMPs—remains operational in CALR-positive patients on ruxolitinib (Figure 3) [12,14].

Triple-negative MPN patients are even less likely to benefit from ruxolitinib, as their disease is not driven by JAK2V617F or a clearly JAK2-dependent pathway. In these patients, upstream drivers may include RAS-MAPK mutations that directly activate ERK1/2 in a JAK-independent manner, making ruxolitinib entirely ineffective against the primary pathogenic signal [1]. TLR2 hyperresponsiveness in triple-negative MPNs, if driven by direct ERK-MAPK pathway mutations, would require alternative targeted approaches such as the introduction of MEK inhibitors.

### 5.4. The TLR2 Axis as a Ruxolitinib-Independent Inflammatory Driver

A critical insight from the DAMP/TLR2 framework is that even in JAK2-positive patients who respond well to ruxolitinib, the TLR2 inflammatory axis may not be fully normalized. Endogenous TLR2/TLR4 ligands (HMGB1, S100A8/A9, NETs) remain elevated in many treated patients, particularly those with higher-risk disease [14], and the DAMP-TLR loop may regenerate independently of JAK-STAT signaling through NF-κB activation and inflammasome-dependent IL-1β release. Plasma IL-1β and IL-6 levels in MF patients correlate closely with DAMP levels [14], suggesting that DAMPs drive cytokine production through TLR-NF-κB pathways that are not targeted by ruxolitinib (Figure 2) [13,14].

This observation may have two important therapeutic implications. First, residual inflammation in patients receiving ruxolitinib may be maintained, at least in part, by TLR2-dependent, JAK-independent mechanisms, providing a potential rationale for combining ruxolitinib with TLR2 antagonists or DAMP-neutralizing agents. Second, in patients who are ineligible for or refractory to ruxolitinib, direct targeting of the TLR2 axis may represent a complementary or alternative anti-inflammatory strategy [14,20]. However, these proposed therapeutic approaches remain hypothetical and require validation in preclinical models and prospective clinical studies.

## 6. Therapeutic Implications: Targeting TLR2 in MPNs

### 6.1. TLR2 Antagonism as a Therapeutic Strategy

Multiple pharmacological approaches to TLR2 inhibition have been developed and validated in preclinical and early clinical settings. OPN-305, a humanized IgG4 monoclonal antibody against TLR2, has undergone Phase I trials in healthy volunteers and demonstrated acceptable safety [7]. C29 (and its derivative ortho-vanillin) is a small-molecule inhibitor of the TLR2 TIR domain, which inhibits both TLR2/1 and TLR2/6 signaling. MMG-11, a selective human TLR2 antagonist with low cytotoxicity, inhibits both TLR heterodimer combinations at nanomolar concentrations [7].

An elegant peptide-based approach—the TLR2 transmembrane domain-derived peptide (TLR2-p)—has been validated in murine DSS-induced colitis as a TLR2 dimerization inhibitor [9]. This peptide physically interacts with TLR2, TLR1, and TLR6 within the membrane, prevents LTA-induced TLR2-TLR6 dimerization (as demonstrated by FRET assay), blocks downstream ERK phosphorylation, and selectively neutralizes the proinflammatory activity of Ly6Chimonocytes without impairing their recruitment [9]. The selective silencing of inflammatory monocyte activity while preserving their migration and homeostatic functions makes TLR2-p conceptually attractive for MPNs, where monocyte hyperactivation drives fibrosis and cytokine excess but monocyte-mediated bone marrow homeostasis needs to be preserved [9,14].

In MPN-specific contexts, TLR2 antagonism could be particularly beneficial in (1) CALR-mutant and triple-negative ET patients with thrombotic risk not addressable by ruxolitinib; (2) MF patients with elevated HMGB1 and S100A8/A9 whose inflammatory state persists despite ruxolitinib; and (3) ET or MF patients experiencing acute thrombotic events or infections that further amplify DAMP release and TLR2 activation [12,14]. The absence of TLR2 overexpression on platelets and monocytes in MPNs—with normal receptor density but hyperactivated downstream signaling—suggests that receptor-blocking antagonists might achieve disproportionate effects in MPN cells that are already primed for low-threshold TLR2 responses [12,14].

### 6.2. DAMP Neutralization as an Upstream TLR2-Targeting Strategy

Given that endogenous DAMPs are the proximate TLR2 (and TLR4) activators in MPNs, neutralizing these ligands upstream of receptor engagement represents an alternative or complementary approach. HMGB1 inhibitors, including recombinant HMGB1 box A (which competitively antagonizes HMGB1 binding to TLR2 and TLR4) and tasquinimod (an S100A9 inhibitor), have demonstrated efficacy in preclinical MPN models [14,20]. Tasquinimod treatment of JAK2V617F murine MPNs reduced bone marrow fibrosis and splenomegaly, implicating the S100A8/A9-TLR axis in disease maintenance [20]. Whether these effects translate to human MPNs, and whether DAMP inhibition can be safely combined with ruxolitinib, are priority questions for clinical investigation.

Anti-NET strategies—including PAD4 inhibitors that prevent citrullination and NET formation, or DNase I that degrades extracellular chromatin—could reduce NET-derived DAMPs in MPN circulation, indirectly attenuating TLR2/TLR4 stimulation [10]. Given that NETs also contribute to thrombosis through direct platelet activation and coagulation cascade priming, anti-NET approaches would simultaneously address both the inflammatory and prothrombotic aspects of MPN pathophysiology [10].

### 6.3. IL-1β Blockade: Targeting TLR2-Downstream Cytokine Effectors

IL-1β, a downstream product of TLR2-NF-κB signaling and NLRP3 inflammasome activation, has recently been identified as a critical driver of MF progression. Two independent murine studies demonstrated that IL-1β promotes clonal expansion of JAK2V617F progenitors and drives bone marrow fibrosis, and that IL-1 receptor antagonism or anti-IL-1β therapy reverses fibrosis in JAK2V617F mouse models [27]. These findings parallel the observation that IL-1β levels in MF patients correlate with DAMP levels and increase progressively with disease stage [14,21]. Canakinumab (anti-IL-1β) or anakinra (IL-1Ra) could thus indirectly attenuate TLR2-driven inflammation in MPNs by blocking a key downstream effector cytokine, while simultaneously addressing TLR2-independent IL-1β sources such as the hyperactivated NLRP3 inflammasome [19,27].

### 6.4. Combination Strategies: Ruxolitinib Plus TLR2 Pathway Inhibition

The complementary mechanisms of ruxolitinib (JAK1/2 inhibition → STAT5/ERK suppression) and TLR2 pathway inhibition suggest that combination strategies could achieve superior anti-inflammatory outcomes in MPNs. Ruxolitinib would address JAK-STAT-driven clonal proliferation and ERK-mediated TLR2 priming, while TLR2 antagonism or DAMP neutralization would interrupt the DAMP–TLR2–NF–κB–cytokine loop that operates independently of JAK signaling [14,24]. Cross-sectional evidence suggests that JAK inhibition does not normalize DAMP levels in many patients [14], indicating that a residual TLR2-dependent inflammatory component persists on ruxolitinib.

For CALR-mutant and triple-negative MPNs, where ruxolitinib has limited molecular efficacy, TLR2-targeted therapies might serve as primary anti-inflammatory agents. The combination of MPL inhibition (for CALR-mutant disease) with TLR2 blockade could address both the proliferative and inflammatory components of pathogenesis more comprehensively than either approach alone. Clinical trials exploring such combinations, guided by biomarker stratification, including TLR2 pathway activity and DAMP levels, are warranted [1,14].

## 7. TLR2 Pathway Components as Biomarkers in MPNs

The DAMP-TLR2 framework suggests several candidate biomarkers for disease stratification, prognostication, and treatment monitoring in MPNs. Plasma HMGB1 and S100A8/A9 levels correlate with disease stage (DIPSS and MIPSS70 risk scores), adverse clinical features (anemia, constitutional symptoms), and inferior overall survival in MF [14]. Combined elevation of both alarmins (present in approximately 34% of MF patients) is associated with particularly poor survival independent of established prognostic scores, with a hazard ratio of 21.2 (95% CI 2.7–163.6), compared to patients with low levels of both DAMPs—suggesting additive or synergistic pathogenic effects [14].

TLR2-triggered platelet functional responses—including P-selectin expression, CD40L, RANTES secretion, and platelet–neutrophil aggregate formation—represent functional biomarkers of platelet inflammatory activation that can be assessed ex vivo using standardized flow cytometry protocols [12]. These parameters differentiate patients with exacerbated TLR2 responses and might predict thrombotic risk or identify patients most likely to benefit from TLR2-targeted therapy. The absence of correlation between TLR2-mediated platelet responses and platelet counts emphasizes that these functional parameters capture qualitative aspects of platelet biology not reflected in routine CBC measurements [12].

Circulating IL-1β and IL-6 levels—which correlate with DAMP levels and disease stage in MF [14]—could serve as pharmacodynamic biomarkers for TLR2 pathway activity in clinical trials of TLR2 antagonists or DAMP inhibitors. Normalization of these cytokines upon treatment would confirm target engagement and suggest biological activity during the intervention [14,25].

## 8. Future Directions and Unanswered Questions

Several important questions remain incompletely addressed and represent priority areas for future investigation. First, the role of TLR2 heterodimer specificity in MPNs has not been systematically examined. Our study suggests that elevated TLR2 is significantly associated with inflammatory response and thrombosis in patients with MPNs, and studies using Pam3CSK4 (TLR2/TLR1) have established TLR2 hyperresponsiveness in ET and MF [12,14]. Thus, the TLR2 pathway may represent an important therapeutic measure in MPNs in that TLR2 acts not only through the JAK2 pathway, but also through the MYD-88-NF-KB pathway, so combining anti-TLR with ruxolitinib may be worthwhile.

Second, the contribution of endosomal TLRs—TLR7, TLR8, and TLR9—to MPN pathogenesis warrants further attention. Endosomal TLRs recognize nucleic acids released from dying cells and NETs, and their activation may contribute to the type I interferon signature observed in some MPN patients [7,22]. Third, the impact of ruxolitinib treatment on DAMP levels and TLR2 pathway activity in MPN patients requires prospective longitudinal assessment. Cross-sectional studies have not demonstrated significant DAMP reduction with ruxolitinib [14], but sequential sampling before and after treatment initiation would be required to determine whether TLR2-dependent inflammation is attenuated or persists during therapy.

Fourth, the NLRP3 inflammasome–TLR2 crosstalk in MPNs warrants dedicated investigation. MF monocytes show hyperactivation of the NLRP3 inflammasome in response to TLR4 ligation and Nigericin, with enhanced IL-1β and IL-18 processing [21]. Whether TLR2 similarly primes NLRP3 inflammasome assembly in MPN monocytes and hematopoietic progenitors—contributing to pyroptotic cell death and ineffective hematopoiesis as described in MDS—is an important mechanistic question [21]. Fifth, clinical trials of TLR2 antagonists or DAMP inhibitors in MPNs are urgently needed. Phase I/II studies assessing safety, pharmacokinetics, target engagement (DAMP and cytokine reduction), and preliminary clinical efficacy in well-defined MPN patient subsets—particularly CALR-mutant and ruxolitinib-refractory patients—would establish whether the robust preclinical rationale translates to clinical benefit [9,14,20].

Finally, regarding whether TLR2 should be considered a disease driver or an inflammatory amplifier, the currently available evidence more strongly supports its role as an inflammatory amplifier. The hypothesis that prolonged inflammatory cytokine signaling promotes the acquisition of JAK2 or other driver mutations remains speculative and will require comprehensive mechanistic and longitudinal studies for validation (Figure 4).

## 9. Conclusions

TLR2 signaling occupies a central position in the thrombo-inflammatory pathophysiology of MPNs. Through its roles in platelet hyperactivation, monocyte cytokine production, platelet–neutrophil aggregate formation, and RANTES-mediated endothelial inflammation, TLR2 serves as a molecular amplifier that transforms the constitutively elevated DAMP environment of MPNs into sustained, clinically meaningful thromboinflammation [12,14]. The endogenous TLR2 ligands present in MPN circulation—NETs and their histone/DNA contents, Hsp27, EDA-fibronectin, HMGB1, and S100A8/A9—provide the foundation for constitutive TLR2 engagement that fuels a self-amplifying inflammatory cycle [10,11,14].

The differential efficacy of ruxolitinib across MPN mutation subtypes is mechanistically anchored, at least in part, in the TLR2-ERK1/2 axis. JAK2V617F constitutively hyperactivates ERK1/2—a shared downstream node of JAK2 and TLR2 signaling—creating molecular priming for exaggerated TLR2 responses that ruxolitinib can partially correct by normalizing JAK2-driven ERK activation [12,24]. CALR mutations, primarily signaling through PI3K/AKT rather than ERK-MAPK, are less efficiently targeted by ruxolitinib, leaving TLR2-mediated thromboinflammation operative despite JAK inhibition [1]. However, no data are currently available regarding the role of the TLR2 pathway; therefore, further studies are needed. In addition, triple-negative MPNs, which lack the classical JAK–STAT driver mutations, may represent the population most likely to benefit from TLR2-targeted rather than JAK-targeted therapies. However, this hypothesis requires further experimental and clinical validation [1,6].

The identification of HMGB1 and S100A8/A9 as elevated, prognostically relevant DAMPs in MF that correlate with disease stage and adverse outcomes [14] opens new avenues for biomarker-guided therapy. Patients with combined elevation of both alarmins face dramatically inferior survival and may represent a population in whom DAMP neutralization or TLR2 blockade could provide the greatest benefit as adjuncts to or replacements for JAK inhibition. Collectively, the evidence could position TLR2 as both a key pathogenic mediator and a tractable therapeutic target in MPNs [2,9,12,14,20].

## Figures and Tables

**Figure 1:**
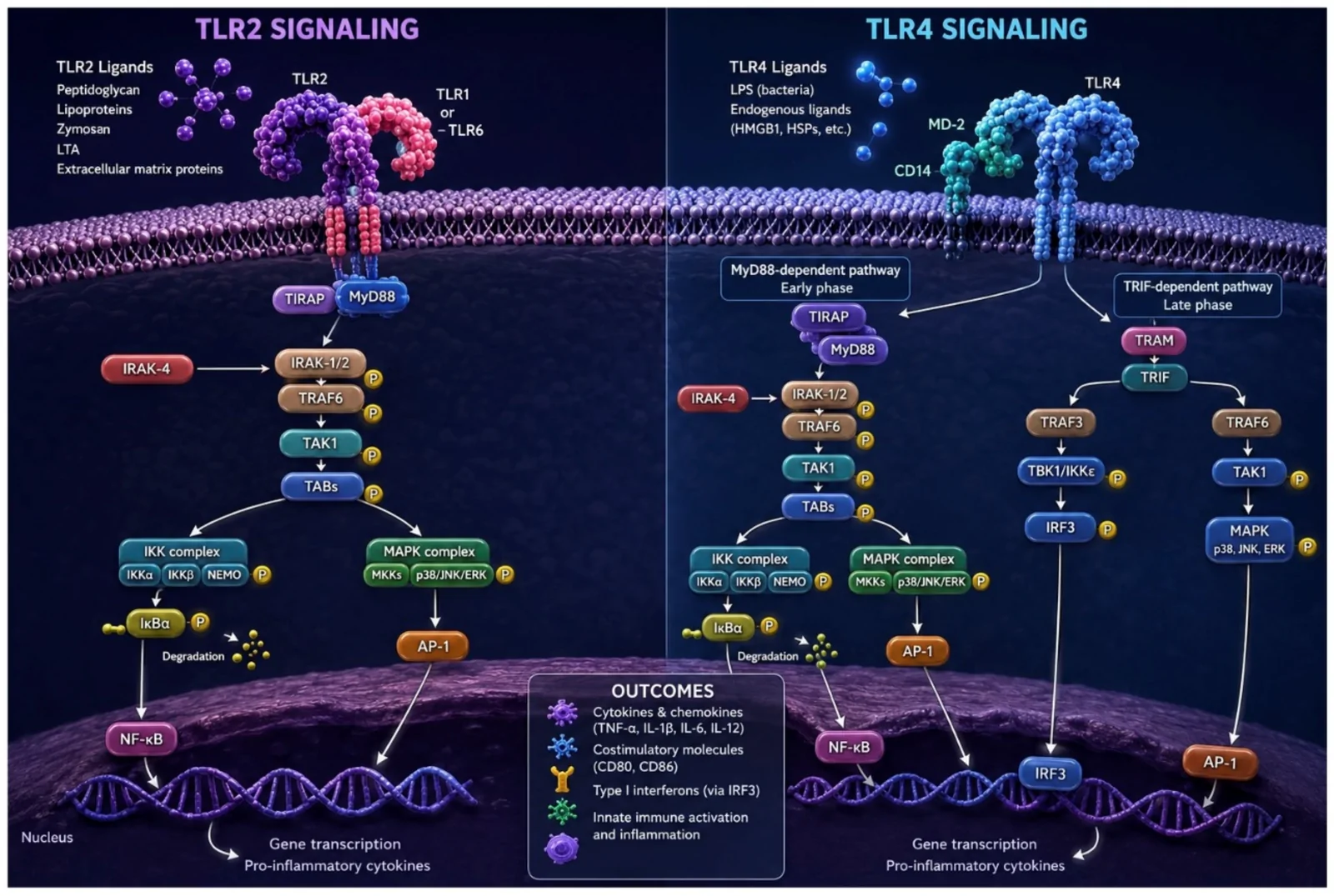
**TLR2 and TLR4 signaling pathways.** TLR2 signaling: Upon ligand-induced dimerization, TLR2 recruits the adaptor MyD88 directly (and, for some ligands, via the bridging adaptor TIRAP/MAL). MyD88 activates IRAK4 and IRAK1/2, which engage TRAF6. TRAF6 stimulates TAK1, leading to phosphorylation and activation of the IKK complex (including IKKβ), subsequent IκBα degradation, and NF-κB nuclear translocation. In parallel, TAK1 initiates MAPK cascades (e.g., p38, JNK, ERK1/2) that enhance inflammatory gene expression, resulting in production of proinflammatory mediators (e.g., TNF-α, IL-1β, IL-6, IL-8, MCP-1). TLR4 signaling: Ligand binding to the TLR4–MD-2 complex promotes receptor dimerization, activating both MyD88- and TRIF-dependent pathways. The MyD88 branch proceeds through TIRAP/MAL (when required) to activate IRAK4/IRAK1/2 → TRAF6 → TAK1 → IKKβ → NF-κB, driving early proinflammatory cytokine transcription. Additionally, TLR4 signals through the TRAM–TRIF axis, culminating in activation of IRF3 and induction of type I interferon responses (e.g., IFN-β) that cooperate with NF-κB-mediated inflammation.

**Figure 2:**
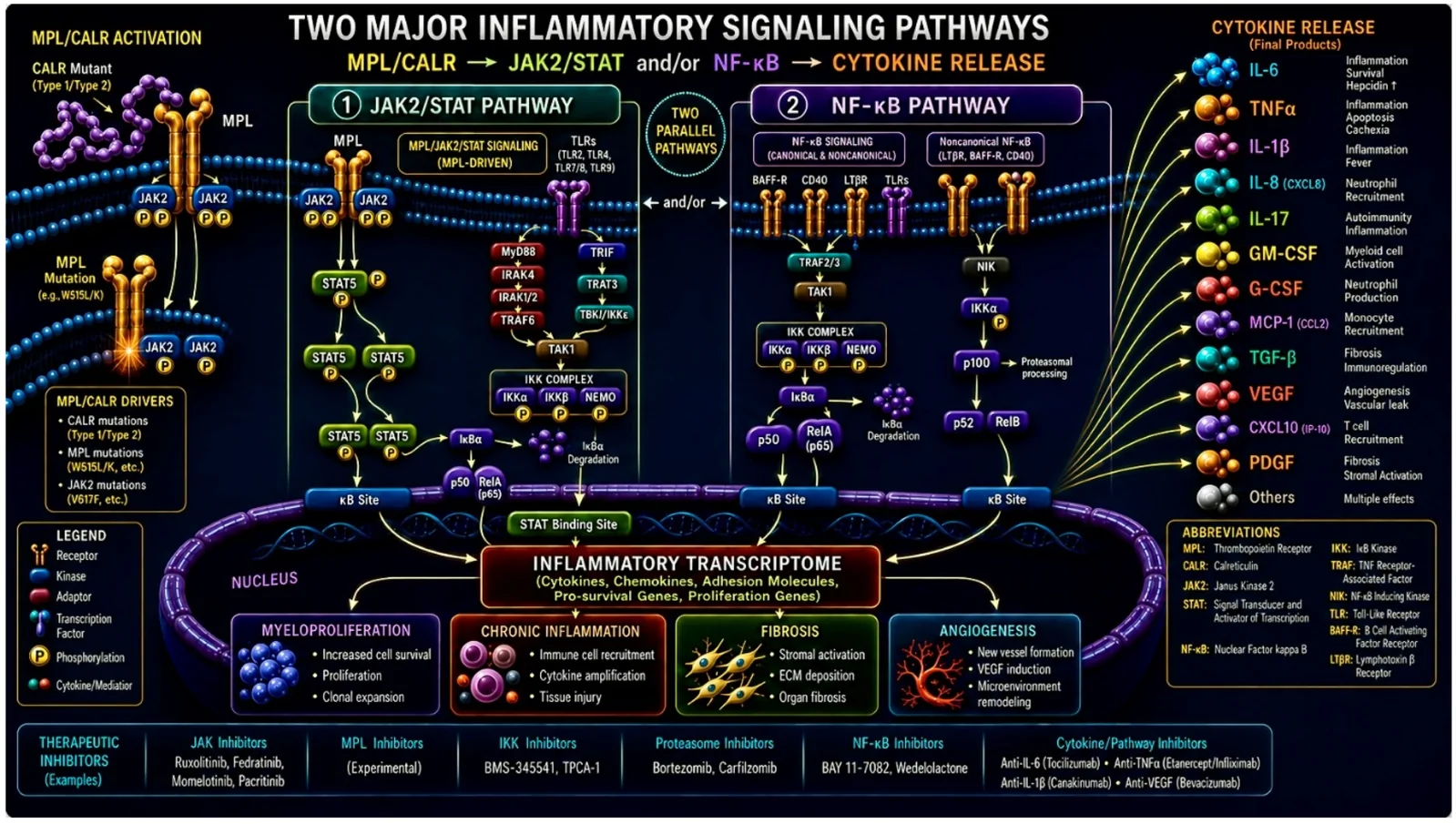
**NF-κB and JAK–STAT signaling pathway.** Two major inflammatory pathways operate in parallel: (1) JAK2/STAT signaling and (2) NF-κB signaling. (1) Along the MPL/CALR axis, signaling initiated by MPL activation (driven by CALR mutations or MPL mutations) or JAK2 mutation leads to JAK2 phosphorylation and activation of STAT5, which translocates to the nucleus and promotes an inflammatory transcriptional program through STAT binding at promoters/enhancers. (2) In parallel, additional upstream cues (including TLR-associated and other canonical/non-canonical inputs) converge on the IκB kinase (IKK) complex, triggering IκBα degradation and NF-κB (p65/RelA) nuclear translocation. Activated NF-κB induces an inflammatory transcriptional signature. Together, these pathways shape the inflammatory transcriptome, including cytokines and chemokines (e.g., IL-6, TNFα, IL-1β, IL-8, IL-17), adhesion molecules, and pro-survival/recruitment genes, culminating in cytokine release and chronic inflammation, with downstream biological effects that can extend to myeloproliferation, fibrosis, and angiogenesis.

**Figure 3:**
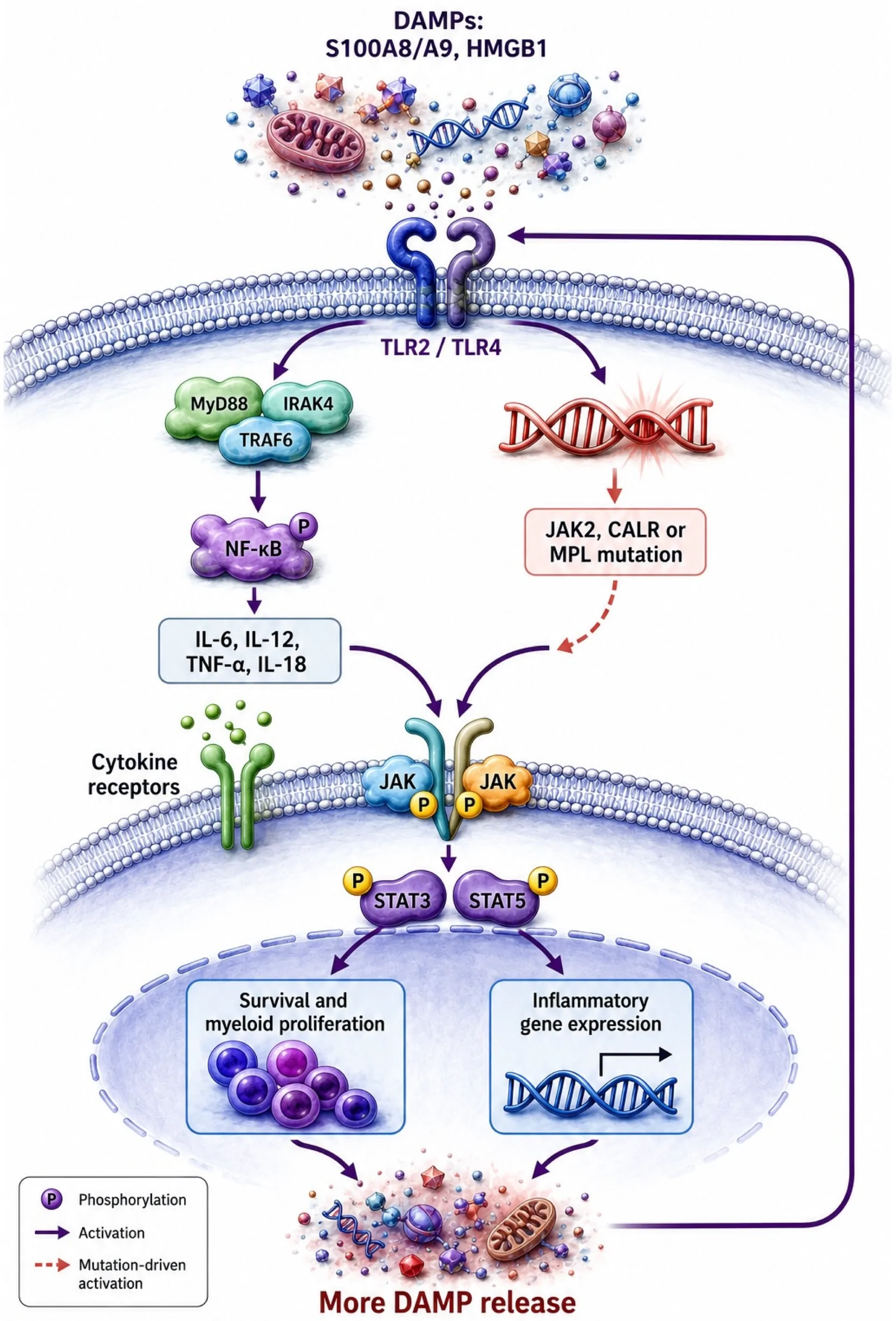
**Thrombo-inflammatory loop.** Released damage-associated molecular patterns (DAMPs) (e.g., S100A8/A9 and HMGB1) activate TLR2/TLR4 on innate immune/stromal cell surfaces, triggering MyD88 → IRAK4 → TRAF6 → NF-κB signaling and inducing proinflammatory cytokines (IL-6, IL-12, TNF-α, IL-18). In parallel, DAMP- and cytokine-receptor signaling converge on JAK/STAT pathways, with cytokines engaging their receptors to drive JAK activation and downstream STAT3/STAT5 phosphorylation. STAT3/STAT5 promote survival and myeloid proliferation while NF-κB- and STAT-dependent programs enhance inflammatory gene expression (including chemo/cytokines), amplifying leukocyte activation and further DAMP release. These bidirectional reinforcement loops couple inflammation to clonal/immune activation, generating a self-sustaining thrombo-inflammatory cycle that promotes progressive dysregulated hematopoiesis and ongoing inflammatory signaling.

**Figure 4:**
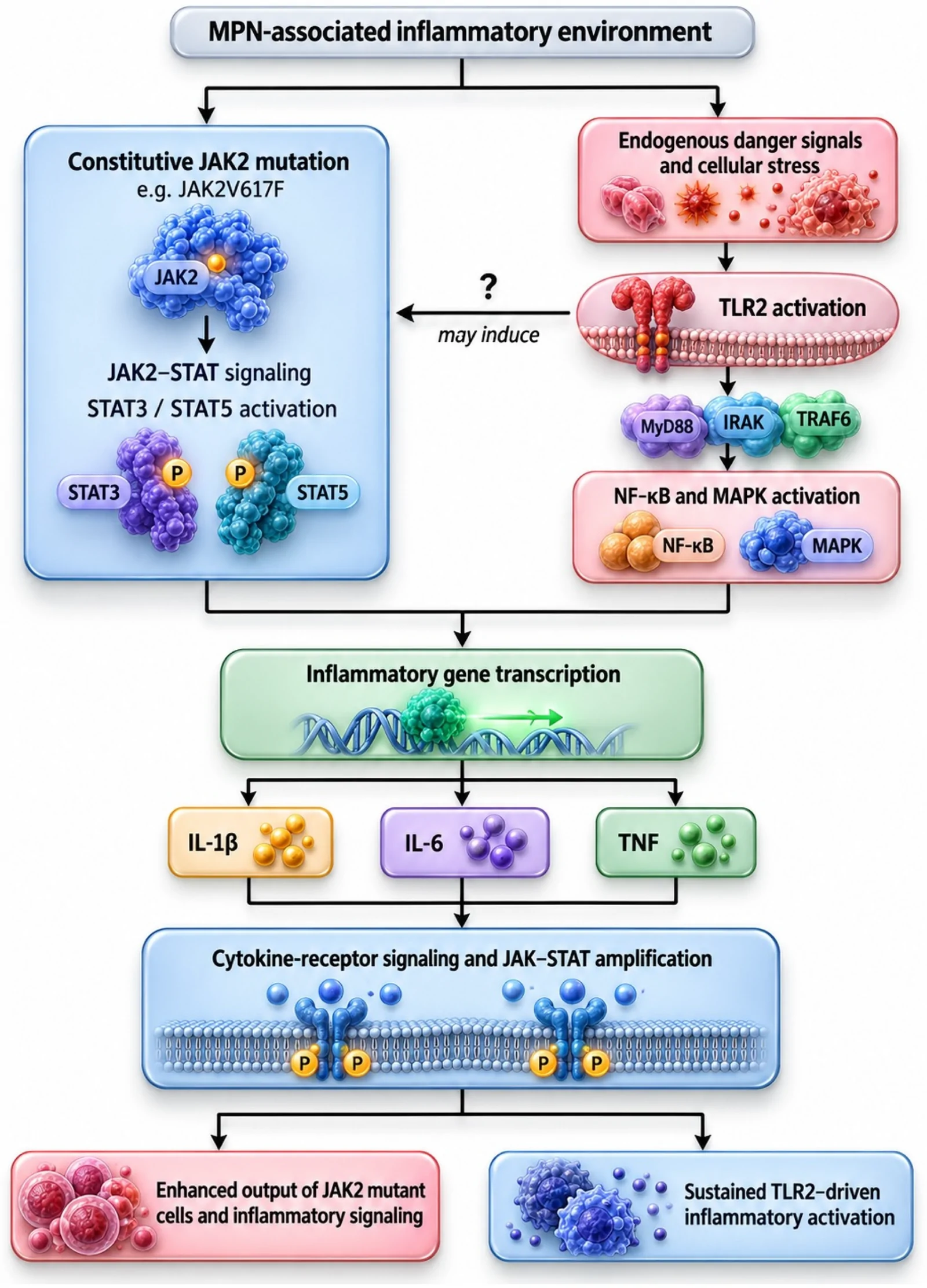
**TLR2 functions more as an inflammatory amplifier than as an initiating driver of disease.** Current evidence supports classifying TLR2 as a candidate inflammatory amplifier rather than an established initiating driver of MPNs. Increased TLR2 expression and responsiveness may enhance the inflammatory and thrombotic consequences of clonal hematopoiesis. However, these associations do not prove that TLR2 initiates clonal disease or is required for its maintenance. Additional studies are needed to determine whether chronic TLR2 activation can induce clonal JAK2 mutations.

## Data Availability

No new data were created or analyzed in this study. Data sharing is not applicable to this article.
